# The Mediating and Moderating Roles of Innovation Climate in the Relationship Between Nurses’ Attitudes Toward Artificial Intelligence, Creative Self‐Efficacy, and Clinical Competence

**DOI:** 10.1155/jonm/1528189

**Published:** 2026-09-27

**Authors:** Ozge Karakaya Suzan, Uğur Gül, Oğuz Koyuncu, Canberk Akdeniz

**Affiliations:** ^1^ Department of Pediatric Nursing, Sakarya University, Sakarya, Türkiye, sakarya.edu.tr; ^2^ Department of Pediatric Nursing, Gümüşhane University, Gümüshane, Türkiye, gumushane.edu.tr; ^3^ Department of Nursing, Yeditepe University, Istanbul, Türkiye, yeditepe.edu.tr

**Keywords:** artificial intelligence, clinical competence, health worker, nursing, organizational culture, self-efficacy

## Abstract

**Background:**

Artificial intelligence (AI) is increasingly integrated into health care; however, how nurses’ attitudes toward AI and creative self‐efficacy are associated with perceived clinical competence within different organizational contexts remains unclear.

**Aim:**

To examine whether perceived climate for innovation statistically mediates the association between nurses’ attitudes toward AI and perceived clinical competence and moderates the association between creative self‐efficacy and perceived clinical competence.

**Methods:**

This cross‐sectional study included 233 nurses recruited online by convenience sampling from hospitals in Türkiye between January and March 2026. Self‐report measures assessed attitudes toward AI, creative self‐efficacy, perceived climate for innovation, and perceived clinical competence. Pearson correlations and PROCESS Models 4 and 1 with 5000 bootstrap samples tested statistical mediation and moderation. Adjusted analyses included age, gender, previous AI‐related training and AI use, educational level, professional experience, and institution type.

**Results:**

Attitudes toward AI were indirectly associated with perceived clinical competence through perceived climate for innovation; the adjusted indirect association remained significant (*B* = 0.0700, SE = 0.0333, 95% CI [0.0173, 0.1453]), consistent with partial statistical mediation. Perceived climate for innovation also moderated the association between creative self‐efficacy and perceived clinical competence after adjustment (interaction *B* = −0.1672, SE = 0.0447, 95% CI [−0.2552, −0.0792]). This positive association became weaker as perceived climate for innovation increased.

**Conclusion:**

Attitudes toward AI, creative self‐efficacy, perceived climate for innovation, and perceived clinical competence were significantly associated, and the statistical mediation and moderation patterns remained after adjustment. The cross‐sectional, self‐report data do not establish temporal or causal mechanisms or effects on objectively measured clinical performance or patient outcomes.

**Implications for Nursing Management:**

Nurse managers may consider supporting AI literacy, safe AI use, team‐based learning, and innovation‐supportive work environments. These strategies should be evaluated prospectively using objective clinical and patient outcomes before their effectiveness is inferred.

**Summary**
• What is already known?
◦ Nurses’ attitudes toward artificial intelligence (AI) influence technology adoption and clinical decision‐making processes.
◦ Creative self‐efficacy is positively associated with innovation, problem‐solving, and clinical competence in nursing practice.
◦ Organizational factors, particularly the climate for innovation, play a critical role in shaping nurses’ performance and innovative behaviors.
◦ Previous studies have generally examined the relationships between AI attitudes, self‐efficacy, and clinical competence separately rather than within an integrated analytical framework.
• What this paper adds?
◦ This study provides a comprehensive model demonstrating both mediating and moderating roles of innovation climate in the relationship between AI attitudes, creative self‐efficacy, and clinical competence.
◦ It reveals that the climate for innovation partially mediates the relationship between nurses’ attitudes toward AI and clinical competence.
◦ The findings show that the climate for innovation moderates the effect of creative self‐efficacy on clinical competence, with the effect diminishing at higher levels of organizational support.
◦ The study highlights that clinical competence is shaped by a dynamic interaction between individual competencies and organizational context, rather than by individual factors alone.
◦ It provides evidence to inform healthcare management strategies for AI integration and innovation‐supportive organizational environments in nursing practice.

## 1. Introduction

The integration of artificial intelligence (AI) technologies into healthcare services has the potential to create a structural transformation in clinical practice by providing critical benefits, such as improving diagnostic processes, reducing workload, and enhancing patient safety [1–4]. Nurses, who occupy a central position in global healthcare systems, play a decisive role in the effective translation of technological transformation into clinical practice [5, 6]. Therefore, identifying the individual and organizational factors that facilitate effective AI adoption has become a strategic priority for nursing management and healthcare organizations [7–10].

Among these individual factors, nurses’ attitudes toward AI play a central role because they influence the acceptance and effective integration of AI technologies into clinical practice [11]. Although nurses generally recognize AI’s potential to improve healthcare quality and efficiency, concerns regarding privacy, ethical issues, algorithmic bias, and the loss of human interaction remain common [7, 8, 12]. These attitudes are shaped by factors, such as perceived usefulness, trust, AI literacy, leadership support, and organizational readiness [7, 13].

Beyond technology acceptance, positive attitudes toward AI have been associated with stronger clinical reasoning, better decision‐making, greater openness to innovation, and increased engagement in creative problem‐solving [1, 14, 15]. However, favorable attitudes alone are insufficient to explain how technological acceptance is translated into competent clinical practice. This process requires individual motivational resources that enable nurses to confidently apply AI within increasingly complex healthcare environments, consistent with the principles of Social Cognitive Theory [7, 16, 17].

From Bandura’s Social Cognitive Theory, creative self‐efficacy (CSE) refers to individuals’ beliefs in their ability to generate novel and useful solutions when confronted with complex problems [18, 19]. Within AI‐supported health care, CSE represents an individual capability associated with how nurses creatively engage with AI‐supported clinical practice. Nurses who view AI positively are more likely to develop confidence in using these technologies creatively and adapting to rapidly changing clinical demands [1, 20].

Higher levels of CSE have consistently been associated with innovative work behavior, stronger problem‐solving skills, enhanced clinical reasoning, and greater confidence in clinical decision‐making [3, 21, 22]. However, Social Cognitive Theory suggests that self‐efficacy develops through continuous interaction with the surrounding environment [18, 19]. Consequently, whether creative confidence translates into competent clinical performance depends substantially on organizational conditions [9, 23].

Innovation climate refers to employees’ shared perceptions that their organization supports experimentation, creative thinking, resource availability, and innovative solutions [24, 25]. Such environments foster learning, collaboration, and openness to change, enabling employees to adapt more effectively to technological innovations [25, 26]. In healthcare settings, a supportive innovation climate reduces uncertainty associated with technological change and facilitates nurses’ confidence in adopting AI within clinical practice [24, 27].

Supportive innovation climates enhance CSE, promote innovative behavior, and contribute to nursing service quality and professional development by facilitating learning, collaboration, and leadership support [9, 23, 25, 28]. Consequently, innovation climate represents not only an organizational characteristic but also a strategic managerial resource for sustaining clinical competence in increasingly digital healthcare environments [9, 23]. Accordingly, innovation climate may serve a dual function within AI‐supported healthcare organizations: as an organizational mechanism through which positive attitudes toward AI are reflected in clinical competence and as a contextual condition that influences the extent to which nurses’ CSE is translated into competent clinical practice [24, 25, 29]. Taken together, these findings highlight the importance of considering both individual and organizational factors when examining nurses’ clinical competence in AI‐supported health care [1, 9, 23].

Clinical competence is a multidimensional construct encompassing clinical reasoning, analytical decision‐making, effective communication, and the ability to adapt to complex clinical situations [30–32]. It is recognized as a key organizational outcome because it is closely associated with nursing care quality, patient safety, and professional development [9, 23]. In AI‐supported health care, competence expectations have expanded to include AI literacy, ethical reasoning, and the safe integration of AI technologies into clinical practice [10, 33–35]. Accordingly, clinical competence is expected to be influenced by both nurses’ individual capabilities and the organizational environment in which care is delivered [1, 7, 9, 14, 21–23].

### 1.1. Conceptual Framework

To better understand the relationships among nurses’ attitudes toward AI, CSE, innovation climate, and clinical competence, this study integrates complementary theoretical perspectives rather than relying on a single theory. Social Cognitive Theory proposes that professional behavior emerges through reciprocal interactions among personal factors, environmental influences, and behavioral outcomes [36, 37]. Within this framework, nurses’ attitudes toward AI represent an important personal factor, whereas CSE represents an individual capability that may contribute to professional performance [38, 39]. Thus, Social Cognitive Theory provides the theoretical basis for examining the relationships among attitudes toward AI, CSE, and clinical competence [36–39].

However, Social Cognitive Theory alone does not fully explain how organizational environments influence this process. The Componential Theory of Creativity proposes that the expression of individual creative potential depends not only on personal motivation and knowledge but also on a supportive organizational environment [40, 41]. Accordingly, nurses with higher CSE are expected to translate their creative confidence into competent clinical performance more effectively when they work in organizations that encourage learning, experimentation, and innovation [9, 23].

From an organizational perspective, Organizational Climate Theory and the Job Demands–Resources (JD‐R) Model conceptualize innovation climate as an organizational resource that supports learning, motivation, innovation, and professional development [42, 43].

These perspectives suggest that organizational resources may influence professional outcomes through two complementary pathways. First, innovation climate may function as an organizational mechanism that helps explain the relationship between nurses’ positive attitudes toward AI and clinical competence. Nurses who hold more positive attitudes toward AI may be more receptive to organizational support for innovation, learning, and the implementation of new technologies, and such supportive conditions may facilitate the effective integration of AI into clinical practice and contribute to higher clinical competence [9, 23]. Second, innovation climate may function as a contextual condition in the relationship between CSE and clinical competence, because nurses with similar levels of CSE may demonstrate different levels of clinical competence depending on whether their organizations provide sufficient support for innovation, learning, and AI implementation [42, 44].

Accordingly, innovation climate is conceptualized in this study as both a mediating and a moderating organizational factor, with each role reflecting a different theoretical function rather than representing the same organizational process. These theoretical perspectives provide distinct yet complementary explanations for the relationships examined in this study. Social Cognitive Theory explains the relationships among attitudes toward AI, CSE, and professional performance, whereas the Componential Theory of Creativity, Organizational Climate Theory, and the JD‐R Model explain how innovation climate may mediate the relationship between attitudes toward AI and clinical competence and shape the extent to which CSE is translated into clinical competence.

Based on these complementary theoretical perspectives, the relationships among nurses’ attitudes toward AI, CSE, innovation climate, and clinical competence are examined within an integrated conceptual framework.

### 1.2. Research Gap and Contribution

Although AI‐related nursing research has expanded rapidly, important theoretical and empirical gaps remain regarding how individual factors and organizational conditions jointly influence nurses’ clinical competence in AI‐supported health care [1, 7, 8]. Existing studies have primarily examined isolated relationships, including nurses’ attitudes toward AI and technology adoption [11, 45], CSE and innovative behavior [3, 24, 46], or innovation climate and organizational outcomes [9, 24, 44]. Consequently, evidence explaining how these individual and organizational factors collectively contribute to nurses’ clinical competence remains limited.

Recent studies have begun to integrate some of these individual‐level variables. For example, Atalla et al. [1] demonstrated significant direct associations among nurses’ attitudes toward AI, CSE, and clinical reasoning competency, indicating that positive attitudes toward AI were associated with greater CSE and better clinical reasoning. While these findings provide important evidence regarding the relationships among individual characteristics, they do not explain how organizational environments may account for or influence these relationships.

Previous studies have examined innovation climate as either a mediating or a moderating variable. However, these roles have been investigated independently rather than within a single conceptual framework [7, 8]. As a result, the simultaneous mediating and moderating roles of innovation climate in the relationships among nurses’ attitudes toward AI, CSE, and clinical competence remain unexplored.

Accordingly, this study aims to examine the mediating and moderating roles of innovation climate in the relationships among nurses’ attitudes toward AI, CSE, and clinical competence. By simultaneously examining innovation climate as both a mediating and a moderating organizational factor, this study extends previous research and provides a more integrated explanation of how individual factors and organizational environments together shape nurses’ clinical competence in AI‐supported health care. The findings are expected to guide healthcare managers in developing organizational strategies that support AI integration, foster innovative work environments, and strengthen nurses’ clinical competence. The research questions of this study are as follows:

1. What are the associations among attitudes toward AI, CSE, innovation climate, and clinical competence in nurses?
2. Does innovation climate mediate the relationship between attitudes toward AI and clinical competence?
3. Does innovation climate moderate the relationship between CSE and clinical competence?

## 2. Methods

### 2.1. Design

This study employed an observational, analytical, cross‐sectional design. Data were collected at a single time point to examine the relationships among nurses’ attitudes toward AI, CSE, perceived climate for innovation, and perceived/self‐reported clinical competence. Mediation and moderation analyses were conducted to examine the indirect and conditional associations among these variables. The study was reported in accordance with the STROBE guidelines for cross‐sectional studies.

### 2.2. Setting, Time, and Participant Recruitment

This online cross‐sectional study was conducted on nurses working in hospitals in different regions of Türkiye who were recruited through an online convenience sampling approach. This approach was chosen for practical reasons to facilitate access to nurses working in various clinical settings across Türkiye. Data were collected between January and March 2026. The Google Forms link remained open during this period and was closed once data collection was complete.

The online survey link was initially distributed to nurses accessible to the researchers and was subsequently shared through hospital‐based WhatsApp groups. In addition, the administrators of nursing‐related Instagram pages were contacted and asked to disseminate the survey link. Nurses who voluntarily accessed the link reviewed the study information and eligibility criteria and proceeded to the questionnaire after providing electronic informed consent. To preserve participant anonymity, hospital names and city‐level identifying information were not collected.

### 2.3. Sample Size and Power for Mediation

The adequacy of the sample size was evaluated using a two‐stage approach. First, based on conventional recommendations for regression‐based models, a medium effect size (*f* ^2^ = 0.15), a significance level of *α* = 0.05, and statistical power (1 − *β*) = 0.95 were adopted in accordance with Jacob Cohen [47]. Under these parameters, the minimum required sample size for the moderation model, which included three predictor variables (CSE, climate for innovation, and the interaction term), was calculated to be approximately 119.

Second, to more precisely assess the mediation effect, a power analysis based on Monte Carlo simulation was conducted [48]. The simulation was based on the path coefficients obtained from the mediation model (path a: *B* = 0.4790, SE = 0.0686; path b: *B* = 0.2414, SE = 0.0736; indirect effect: *B* = 0.1156). The simulation results, conducted with 5000 replications and *α* = 0.05, indicated that the minimum required sample size to detect the indirect effect with 95% power ranged between approximately 130 and 150. Accordingly, the sample of 233 nurses included in this study was considered sufficient to reliably test direct, indirect, and interaction effects.

The inclusion criteria for participants were as follows: (i) being employed as a nurse in a hospital, (ii) being able to read and understand Turkish, (iii) owning a smartphone or computer, (iv) having Internet access, and (v) voluntarily agreeing to participate in the study. The exclusion criteria were as follows: (i) withdrawal from the study at any stage.

### 2.4. Data Collection Instruments

#### 2.4.1. Descriptive Information Form

The researchers developed an 11‐item questionnaire for nurses. Of these, eight items were designed to collect sociodemographic information, including age, gender, marital status, unit of work, educational background, years of professional experience, type of institution, shift schedule, and nursing position. The remaining three items focused on nurses’ training in AI and their experience with AI use.

#### 2.4.2. Artificial Intelligence Attitude Scale for Nurses (AIASN)

The AIASN, developed by Yıldırım and Karaman [49], consists of a total of 32 items and four subdimensions. The subdimensions are as follows: Nursing Care (Items 1–14), Organization (Items 15–20), Ethics (Items 21–26), and Artificial Intelligence Readiness (Items 27–32). The scale is rated on a 5‐point Likert scale ranging from “*1 = Strongly disagree*” to “*5 = Strongly agree*.” Higher scores on the scale indicate more positive attitudes of nurses toward AI. In the Nursing Care subdimension, Items 4, 13, and 14, as well as all items in the Ethics subdimension, are reverse‐coded. The overall Cronbach’s alpha coefficient of the scale is 0.925, with subdimension values ranging between 0.913 and 0.960. In this study, the Cronbach’s alpha coefficient for the total AIASN was *α* **= **0.798.

#### 2.4.3. Creative Self‐Efficacy Scale (CSES)

The CSES is the Turkish adaptation of the original English version developed by Tierney and Farmer [50]. Its Turkish validity and reliability were established by Atabek [51]. The CSES measures employees’ beliefs in their ability to be creative in their work. The scale consists of three items rated on a 5‐point Likert scale, with responses ranging from “*1 = Strongly disagree*” to “*5 = Strongly agree*.” The total score ranges from 3 to 15, with higher scores indicating stronger CSE. The Cronbach’s alpha coefficient of the scale has been reported as 0.877. In this study, the Cronbach’s alpha coefficient for the CSES was *α* = 0.807.

#### 2.4.4. Competence Scale for Clinical Nurses (CSFCN)

The CSFCN, developed by Kao et al. [52] and adapted into Turkish by Köroğlu et al. [53], consists of 18 items. In the validity and reliability process of the scale, content, face, and construct validity were established, followed by the calculation of Cronbach’s alpha coefficients. The content validity index of the scale was determined as 0.87 based on the evaluation of five experts. The factor structure of the scale, identified through exploratory factor analysis, comprises three subdimensions: clinical care skills (Items 1–9), work engagement (Items 10–14), and patient‐centered and ethical considerations (Items 15–18). The CSFCN assesses nurses’ self‐reported/perceived clinical competence rather than objectively observed clinical performance.

The scale is rated on a 7‐point Likert scale ranging from “*1 = Not at all appropriate*” to “*7 = Completely appropriate*,” with higher scores indicating higher levels of clinical competence. In the study by Kao et al. [52], the Cronbach’s alpha values were reported as 0.91 for the total scale, 0.90 for clinical care skills, 0.84 for work engagement, and 0.89 for patient‐centered and ethical considerations. In this study, the Cronbach’s alpha coefficient for the total CSFCN was *α* **= **0.923.

#### 2.4.5. Scale of Climate for Innovation (SCI)

The SCI, developed by Scott and Bruce [54] and adapted into Turkish by Sönmez et al. [55], consists of a total of 14 items across three subdimensions and is rated on a 5‐point Likert scale. All items in the “support for innovation” subdimension are scored directly (*1 = strongly disagree*, *5 = strongly agree*), and higher mean scores indicate a higher level of organizational support for innovation. All items in the “innovation barriers” subdimension are reverse‐scored (*1 = strongly agree*, *5 = strongly disagree*), and higher mean scores indicate fewer organizational barriers to innovation. All items in the “resource supply” subdimension are scored directly (*1 = strongly disagree*, *5 = strongly agree*), and higher mean scores indicate that the organization provides resources to support innovation. The Cronbach’s alpha coefficient for the overall scale was reported as 0.824, with subdimension values ranging between 0.734 and 0.849. In this study, the Cronbach’s alpha coefficient for the total SCI was *α* = 935.

### 2.5. Covariate Variables

Based on previous literature [1, 3, 7, 9, 11], covariate‐adjusted sensitivity analyses were conducted for both the mediation and moderation models. Age, gender, previous AI‐related training, previous AI use, educational level, professional experience, and institution type were included as covariates. Categorical covariates with more than two categories were dummy‐coded before being entered into the PROCESS models. Reference Categories: Bachelor’s degree for educational status, 0–5 years for clinical experience, and state hospital for type of hospital.

### 2.6. Data Collection

The nurse information form and scales were structured as an online questionnaire using Google Forms. All questions in the online form were set as mandatory; therefore, participants were unable to submit the questionnaire without responding to every item. Consequently, there were no missing data or incompletely completed questionnaires. To prevent duplicate submissions, the “Limit to one response” feature in Google Forms was enabled, allowing only one questionnaire submission per user account.

The study announcement and questionnaire link were shared through Instagram pages that posted content relevant to the target population and WhatsApp groups that included individuals from the target group. The Instagram pages and WhatsApp groups were selected purposively based on their accessibility to the target population and their relevance to the research topic and intended participant group. The questionnaire link was shared after obtaining permission from the administrators of the respective pages or groups. Administrators of some institutions also supported the dissemination of the study announcement through appropriate communication channels. However, this process did not involve formal correspondence with the institutions or an official institutional distribution of the questionnaire. The institutional administrators merely facilitated the voluntary sharing of the study announcement and online questionnaire link within relevant groups. Participants were then directed to the Google Forms questionnaire by clicking on the link. The purpose and scope of the study, conditions of voluntary participation, and data confidentiality policy were clearly presented to participants on the first page of the online survey. Participants were informed, before clicking the “Continue” button, that participation was entirely voluntary, that they could withdraw at any time, that no personal information (e.g., name, surname, IP address, and email) would be collected, that all responses would be stored anonymously, and that the data would be used solely for scientific purposes. To ensure anonymity, the online questionnaire was configured not to collect participants’ names, surnames, email addresses, IP addresses, or other direct personal identifiers. Participants were not asked to provide the name of their hospital or city, and individual responses could not be linked to a specific participant or institution. To ensure data confidentiality, the dataset was stored in a password‐protected electronic file accessible only to the research team. The findings were analyzed and reported in aggregate form, and no individual‐level information was disclosed.

An online informed consent form was also included on this page, and participants were allowed to proceed to the questionnaire only after providing consent. The questionnaire consisted of five main sections. The first section included participants’ demographic information, while the subsequent sections presented the scales to be used in sequence. The average time required to complete the questionnaire was approximately 20 min.

### 2.7. Statistical Analysis

The data were analyzed using IBM SPSS Statistics (Version 26.0). In the evaluation of the study data, categorical variables were summarized using frequency and percentage distributions, while continuous variables were described using mean, standard deviation, minimum, and maximum values. The dependent variable of the study was perceived/self‐reported clinical competence, whereas the independent variables were nurses’ attitudes toward AI and their CSE. The climate for innovation variable was treated as both a mediator and a moderator in separate analyses. Pearson’s correlation analysis was conducted to examine the relationships among variables. Mediation and moderation analyses were performed using the PROCESS macro. Model 4 was used for mediation analysis, and Model 1 was used for moderation analysis. The significance of indirect effects was assessed using 5000 bootstrap samples. The statistical results were evaluated based on 95% confidence intervals in terms of clinical significance. The internal consistency of the scales was assessed using Cronbach’s alpha coefficient. Harman’s single‐factor test was conducted to assess the potential risk of common method bias [56]. All items from the main study scales were entered into an unrotated principal component analysis, and the variance explained by the first factor was examined. The first factor explained 17.59% of the total variance, which was well below commonly used threshold values, suggesting that common method bias was unlikely to substantially affect the study findings. Statistical significance was set at *p* < 0.05.

### 2.8. Ethical Considerations

Ethical approval was obtained from the Sakarya University Ethics Committee on December 29, 2025 (Approval No. E‐61923333‐050.99‐546697). The study was conducted in accordance with the principles of the Declaration of Helsinki. Before accessing the questionnaire, participants were provided with information about the purpose and scope of the study, voluntary participation, confidentiality, and the intended use of the data. Participants could discontinue the questionnaire at any time before submitting their responses without any consequences. Electronic informed consent was obtained before participation, and access to the questionnaire was permitted only after consent had been provided. The questionnaire and the data obtained were used solely for the purposes of this study, and the findings were reported in aggregate form. As recruitment was conducted online at the individual level and no hospitals were designated as formal study sites, hospital‐specific institutional permission was not applicable to the recruitment procedure.

## 3. Results

According to the study findings, females constituted the majority of the sample (*n* = 167, 71.67%). A large proportion of participants were married (*n* = 111, 47.64%). More than half of the participants held a bachelor’s degree (*n* = 163, 69.96%), and 42.92% had 0–5 years of professional experience. In terms of working pattern, most participants worked in shifts (*n* = 153, 65.67%). Nearly half of the participants were employed in public hospitals (*n* = 116, 49.79%). The majority of participants (*n* = 215, 92.27%) reported that they had not received training in the use of generative AI tools. However, 65.67% indicated that they used generative AI tools. Regarding the type of AI tools used, the most common was the use of only ChatGPT (*n* = 111, 50.00%). The mean age of the participants was 32.39 ± 7.48 years (Table 1).

**TABLE 1:** Descriptive characteristics of the participants (*n* = 233).

Variables							*n*	%
Gender								
Male							66	28.33
Female							167	71.67
Marital status								
Single							122	52.36
Married							111	47.64
Educational status								
High school							2	0.8
Associate degree							5	2.15
Bachelor’s degree							163	69.96
Master’s degree							55	23.61
Doctoral degree							8	3.43
Clinical experience								
0–5 years							100	42.92
6–10 years							55	23.61
11–15 years							27	11.59
15 years or more							51	21.89
Work schedule								
Shift work							153	65.67
Day shift only							58	24.89
Night shift only							22	9.44
Type of hospital								
City hospital							46	19.74
University hospital							50	21.46
State hospital							116	49.79
Private hospital							21	9.01
Training in the use of generative artificial intelligence tools								
Yes							18	7.73
No							215	92.27
Use of generative artificial intelligence tools								
Yes							153	65.67
No							80	34.33
Artificial intelligence tools used^∗^								
Only ChatGPT							111	50.00
Only Gemini							10	4.50
Only Grok							1	0.45
ChatGPT and Gemini							56	25.23
ChatGPT and Copilot							10	4.50
ChatGPT, Gemini, and Grok							7	3.15
ChatGPT, Gemini, and Claude							23	10.36
ChatGPT, Gemini, and Kling ai							2	0.90
ChatGPT, Copilot, Gemini, and Grok							2	0.90

**Variables**	**M**	**SD**	***n***	**Max**	**Min**	**95% CI**	**Skewness**	**Kurtosis**

Age	32.39	7.48	233	52.00	23.00	[31.421, 33.352]	0.83	−0.31
AIASN	113.03	9.56	233	141.00	84.00	[111.800, 114.268]	−0.12	0.22
CSES	11.21	1.91	233	15.00	6.00	[10.969, 11.461]	0.13	−0.71
SCI	40.99	10.96	233	64.00	14.00	[39.576, 42.406]	0.33	−0.32
CSFCN	104.80	15.02	233	126.00	55.00	[102.864, 106.741]	−1.44	1.93

*Note:* M: mean; *n*: sample size; %: percentage; Max: maximum; Min: minimum; 95% CI: lower and upper bounds of the 95% confidence interval.

Abbreviations: AIASN, Artificial Intelligence Attitude Scale for Nurses; CSES, Creative Self‐Efficacy Scale; CSFCN, Competence Scale for Clinical Nurses; SCI, Scale of Climate for Innovation; SD, standard deviation.

^∗^Multiple responses were allowed.

The mean total scores were 113.03 ± 9.56 for the AIASN, 11.21 ± 1.91 for the CSES, 40.99 ± 10.96 for the SCI, and 104.80 ± 15.02 for the CSFCN.

Pearson’s correlation analysis was conducted to examine the relationships among the AIASN, CSES, SCI, and CSFCN. A significant positive correlation was observed between AIASN and CSES (*r* = 0.61), indicating a large effect size. A significant positive correlation was also found between AIASN and SCI (*r* = 0.42). In addition, AIASN and CSFCN were positively and significantly correlated (*r* = 0.65), indicating a large effect size. A significant positive correlation was observed between CSES and SCI (*r* = 0.34), reflecting a moderate effect size. Similarly, CSES and CSFCN showed a significant positive correlation (*r* = 0.47), indicating a moderate effect size. A significant positive correlation was also found between SCI and CSFCN (*r* = 0.42), corresponding to a moderate effect size. Pearson’s correlation results are presented in Table 2.

**TABLE 2:** Pearson’s correlation coefficients between main variables (*n* = 233).

Variable	AIASN	CSES	SCI	CSFCN
1. AIASN	—			
2. CSES	0.61^∗∗^	—		
3. SCI	0.42^∗∗^	0.34^∗∗^	—	
4. CSFCN	0.65^∗∗^	0.47^∗∗^	0.42^∗∗^	—

Abbreviations: AIASN, Artificial Intelligence Attitude Scale for Nurses; CSES, Creative Self‐Efficacy Scale; CSFCN, Competence Scale for Clinical Nurses; SCI, Scale of Climate for Innovation.

^∗∗^Indicates that the correlation is significant at the 0.01 level (two‐tailed).

A mediation analysis was conducted to examine whether the SCI mediates the relationship between the CSES and the CSFCN. Variance inflation factors (VIFs) were calculated to assess multicollinearity, with a threshold value of less than 5 considered acceptable [57]. The VIF values were 1.10 for SCI and 1.10 for CSES, indicating no evidence of multicollinearity.

Mediation was examined based on indirect and direct effects using bootstrapping (*N* = 5000) with percentile‐based confidence intervals. The results of the simple mediation analysis are presented in Table 3. The mediation analysis yielded several significant findings. The AIASN was positively and significantly associated with SCI (*B* = 0.4790, SE = 0.0686, *t* = 6.99, *p* < 0.001). SCI was also found to be a significant positive direct predictor of CSFCN (*B* = 0.2414, SE = 0.0736, *t* = 3.28, *p* < 0.001). Additionally, AIASN directly and positively predicted CSFCN (*B* = 0.9101, SE = 0.0844, *t* = 10.78, *p* < 0.001). Regarding indirect effects, AIASN was found to significantly predict CSFCN through SCI (*B* = 0.1156, 95% CI [0.0484, 0.1875]). This finding indicates that SCI plays a significant mediating role in the relationship between AIASN and CSFCN.

**TABLE 3:** Simple mediation analysis (Model 4) results predicting CSFCN (*n* = 233).

Pathway	*B*	SE	*t*	*p*	LLCI	ULCI
Unadjusted model						
Direct effects						
AIASN ⟶ SCI	0.4790	0.0686	6.987	*p* < 0.001	0.3439	0.6140
SCI ⟶ CSFCN	0.2414	0.0736	3.280	*p* < 0.001	0.0964	0.3865
AIASN ⟶ CSFCN	0.9101	0.0844	10.781	*p* < 0.001	0.7438	1.0764
Indirect effects						
AIASN ⟶ SCI ⟶ CSFCN	0.1156	0.0353	—	—	0.0484	0.1875
Total effect (direct + indirect)	1.0257	0.0783	13.0996	*p* < 0.001	0.8715	1.1800
Total effect model: (AIASN ⟶ CSFCN, mediator excluded): *R* ^2^ = 0.426, *F*(1, 231) = 171.60, *p* < 0.001.						
Final mediation model: (AIASN + SCI ⟶ CSFCN): *R* ^2^ = 0.452, *F*(2, 230) = 94.80, *p* < 0.001.						
Adjusted model						
Direct effects						
AIASN ⟶ SCI	0.3673	0.0808	4.546	*p* < 0.001	0.2081	0.5266
SCI ⟶ CSFCN	0.1905	0.0777	2.452	0.015	0.0374	0.3436
AIASN ⟶ CSFCN	0.8641	0.0968	8.931	*p* < 0.001	0.6734	1.0548
Indirect effect						
AIASN ⟶ SCI ⟶ CSFCN	0.0700	0.0333	—	—	0.0173	0.1453
Total effect (direct + indirect)	0.9340	0.0935	9.988	*p* < 0.001	0.7497	1.1184
Total effect model: (AIASN ⟶ CSFCN, mediator excluded): *R* ^2^ = 0.473, *F*(15, 217) = 12.96, *p* < 0.001						
Final mediation model: (AIASN + SCI ⟶ CSFCN): *R* ^2^ = 0.487, *F*(16, 216) = 12.81, *p* < 0.001.						

*Note:* LLCI/ULCI: lower and upper bounds of the 95% confidence interval; *B*: unstandardized regression coefficient; *t*: *t* statistic; *p*: significance level (*p* value).

Abbreviations: AIASN, Artificial Intelligence Attitude Scale for Nurses; CSFCN, Competence Scale for Clinical Nurses; SCI, Scale of Climate for Innovation; SE, standard error.

The total effect, encompassing both direct and indirect effects, was also statistically significant (*B* = 1.0257, SE = 0.0783, *t* = 13.10, *p* < 0.001). This result suggests that AIASN increases CSFCN both directly and indirectly through SCI. Model fit indices were also found to be statistically significant. When the mediator variable was not included (total effect model), 42.6% of the total variance was explained (*R* ^2^ = 0.426, *F*(1, 231) = 171.60, *p* < 0.001). When the mediator variable (SCI) was included in the model, the explained variance increased to 45.2% (*R* ^2^ = 0.452, *F*(2, 230) = 94.80, *p* < 0.001). Overall, these findings indicate that SCI plays a significant mediating role in the relationship between AIASN and CSFCN. The mediation model is presented in Figure 1.

**FIGURE 1:**
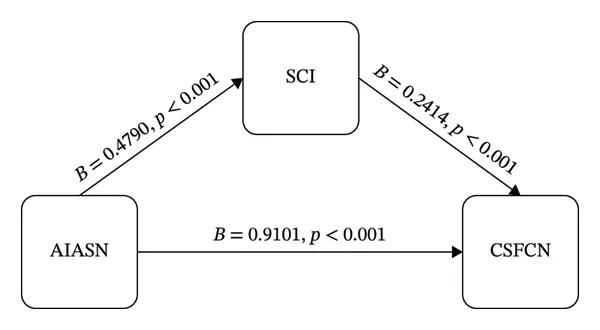
The mediating effect of the Scale of Climate for Innovation (SCI) in the relationship between the Artificial Intelligence Attitude Scale for Nurses (AIASN) and the Competence Scale for Clinical Nurses (CSFCN).

In the unadjusted mediation model, SCI partially mediated the relationship between AIASN and CSFCN. The indirect effect was significant (*B* = 0.1156, SE = 0.0353, 95% CI [0.0484, 0.1875]). In the covariate‐adjusted sensitivity analysis, the indirect effect was attenuated but remained significant (*B* = 0.0700, SE = 0.0333, 95% CI [0.0173, 0.1453]). The direct effect of AIASN on CSFCN also remained significant after adjustment (*B* = 0.8641, SE = 0.0968, *p* < 0.001), indicating partial mediation.

The results of the moderation analysis (Model 1) indicated that the overall model was statistically significant (*R* ^2^ = 0.33, *F*(3, 229) = 37.87, *p* < 0.001), explaining 33% of the variance in the dependent variable.

Examination of the main effects revealed that the CSES had a significant and positive effect on the dependent variable (*B* = 2.979, SE = 0.452, *t* = 6.59, *p* < 0.001). The SCI was also found to be a significant positive predictor of the CSFCN (*B* = 0.487, SE = 0.0827, *t* = 5.89, *p* < 0.001). Regarding the interaction effect, the interaction between CSES and SCI was statistically significant and negative (*B* = −0.139, SE = 0.0414, *t* = −3.37, *p* < 0.001). This result indicates that SCI significantly moderates the relationship between CSES and the dependent variable. Overall, the findings demonstrate that the effect of CSES on the dependent variable varies depending on the level of SCI, thereby supporting the proposed moderation model.

After controlling for age, gender, previous AI‐related training, previous AI use, educational level, professional experience, and institution type, the overall moderation model remained statistically significant (*R* ^2^ = 0.388, *F*(17, 215) = 8.01, *p* < 0.001). CSES remained a significant positive predictor of CSFCN (*B* = 2.194, SE = 0.523, *t* = 4.19, *p* < 0.001), and SCI was also positively associated with CSFCN (*B* = 0.418, SE = 0.087, *t* = 4.82, *p* < 0.001). The interaction between CSES and SCI remained statistically significant and negative (*B* = −0.167, SE = 0.045, *t* = −3.74, *p* < 0.001), indicating that the moderating role of SCI was retained after adjustment for the selected covariates (Table 4). The moderating effect is illustrated in Figure 2.

**TABLE 4:** Simple moderation analysis (Model 1) results predicting CSFCN (*n* = 233).

Predictor	*B*	SE	*t*	*p*	LLCI	ULCI
Unadjusted model						
(Intercept)	105.780	0.860	122.966	*p* < 0.001	104.085	107.475
Main effect						
CSES	2.979	0.452	6.587	*p* < 0.001	2.088	3.870
SCI	0.487	0.0827	5.887	*p* < 0.001	0.324	0.650
Interaction effects						
CSES ∗ SCI	−0.139	0.0414	−3.365	*p* < 0.001	−0.221	−0.0577
Overall model: *R* ^2^ = 0.33, *F*(31, 229) = 37.87, *p* < 0.001						
Adjusted model						
(Intercept)	96.6330	7.3249	13.192	*p* < 0.001	82.1951	111.0709
CSES	2.1940	0.5233	4.193	*p* < 0.001	1.1626	3.2254
SCI	0.4180	0.0868	4.818	*p* < 0.001	0.2470	0.5890
Interaction effects						
CSES ∗ SCI	−0.1672	0.0447	−3.744	*p* < 0.001	−0.2552	−0.0792
Overall model: *R* ^2^ = 0.3876, *F*(17, 215) = 8.01, *p* < 0.001						

*Note:* LLCI/ULCI: lower and upper bounds of the 95% confidence interval; B: unstandardized regression coefficient; *β*: standardized regression coefficient; *t*: *t* statistic; *p*: significance level (*p* value).

Abbreviations: CSES, Creative Self‐Efficacy Scale; CSFCN, Competence Scale for Clinical Nurses; SCI, Scale of Climate for Innovation; SE, standard error.

**FIGURE 2:**
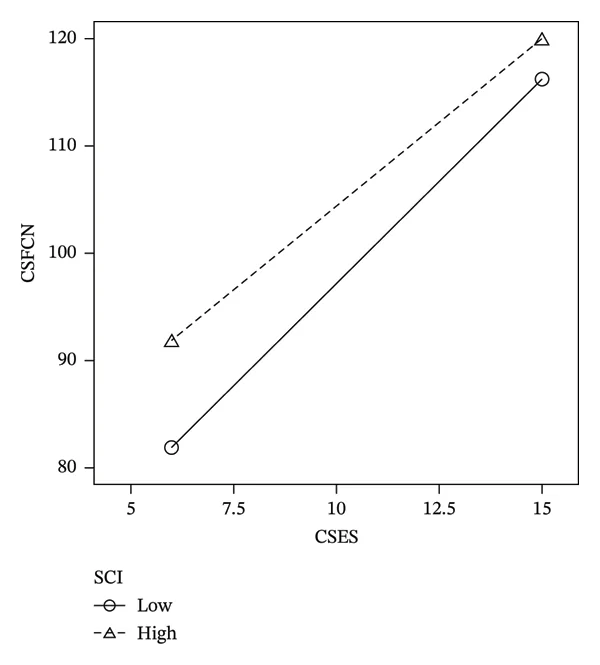
The moderating role of the Scale of Climate for Innovation (SCI) in the relationship between the Creative Self‐Efficacy Scale (CSES) and the Competence Scale for Clinical Nurses (CSFCN).

Since the interaction effect was found to be significant in the moderation analysis, a simple slopes analysis was conducted to examine how the effect of the CSES on the CSFCN varies at different levels of the moderator (SCI fixed at its mean, one standard deviation above the mean, and one standard deviation below the mean). When SCI was fixed at 30.03, the coefficient of CSES was significant (*B* = 4.51, *p* < 0.001). When SCI was fixed at 40.99, the coefficient of CSES remained significant (*B* = 2.98, *p* < 0.001). When SCI was fixed at 51.95, the coefficient of CSES was still significant (*B* = 1.45, *p* = 0.023). However, when SCI was fixed at 54.00, the coefficient of CSES was not statistically significant (*B* = 1.16, *p* > 0.05). These findings indicate that as the level of SCI increases, the relationship between CSES and CSFCN weakens. The results of the simple slopes analysis are presented in Table 5.

**TABLE 5:** Simple slopes analysis for SCI moderating the relationship between CSES and CSFCN.

SCI values	*B*	SE	*t*	*p*	LLCI	ULCI
30.03	4.51	0.65	6.95	< 0.001	3.23	5.78
40.99	2.98	0.45	6.59	< 0.001	2.09	3.87
51.95	1.45	0.63	2.29	0.023	0.20	2.70
54.00	1.16	0.66	1.67	0.09	−0.20	2.53

*Note:* LLCI/ULCI: lower and upper bounds of the 95% confidence interval; *B*: unstandardized regression coefficient; *t*: *t* statistic; *p*: significance level (*p* value).

Abbreviations: CSES, Creative Self‐Efficacy Scale; CSFCN, Competence Scale for Clinical Nurses; SCI, Scale of Climate for Innovation; SE, standard error.

## 4. Discussion

This study examined the mediating role of the climate for innovation in the relationship between nurses’ attitudes toward AI and perceived clinical competence, as well as the moderating role of the climate for innovation in the relationship between CSE and perceived clinical competence. The results showed that SCI partially mediated the relationship between AIASN and perceived clinical competence. SCI also moderated the relationship between CSES and perceived clinical competence, with this relationship becoming weaker at higher levels of SCI. Both effects remained statistically significant after adjustment for demographic, professional, and AI‐related variables. However, because of the cross‐sectional design, these findings indicate associations rather than causal relationships.

A recent meta‐analysis synthesizing AI awareness, attitudes, and use among nursing students, clinical nurses, and nurse academics reported an AI use rate of 56% among clinical nurses and 67% across all nursing roles [58]. In this study, 65.67% of nurses reported using generative AI. This rate was higher than the global clinician rate and numerically similar to the overall rate reported in the meta‐analysis. However, this comparison should be interpreted cautiously because the studies may differ in their definitions of AI use, participant roles, data collection periods, and types of AI technologies assessed. In Türkiye, Tuncer and Tuncer [59] reported that 72.2% of nurses had not used an AI program and 51% had no knowledge of AI. The higher use rate observed in this study may reflect differences between the samples and the increasing availability of generative AI tools. This interpretation is also consistent with reports indicating a recent increase in AI use among clinicians [60]. Nevertheless, the questionnaire did not distinguish between clinical, educational, and personal use of generative AI; therefore, the reported rate should not be interpreted as the prevalence of AI use specifically in clinical practice.

Although AI may support clinical decision‐making [14, 61], care planning, and healthcare management [62], reported use alone does not indicate whether these tools are used effectively, safely, or for clinical purposes. A substantial proportion of clinical AI applications also remain at the testing stage or are discussed mainly in terms of their potential benefits [63]. An important consideration is therefore whether nurses use AI appropriately, critically, ethically, and in accordance with the clinical context. In this study, only 7.73% of participants had received formal training, although 65.67% reported using generative AI. This marked difference suggests that the use of generative AI among nurses may not be adequately supported by formal and institutional training. Previous research similarly indicates that formal AI education remains limited and that learning frequently occurs through informal and extracurricular channels [64]. Undergraduate and postgraduate nursing programs may therefore consider including competencies related to the accurate, ethical, and effective use of AI in their curricula [58]. Such education could address not only technical skills but also concerns related to human interaction, professional nursing roles, care ethics, algorithmic bias [65], and controllability [66, 67]. Educational programs may also address nurses’ attitudes and concerns about AI rather than focusing only on technical skills [68].

The mean AIASN score was 113.03 ± 9.56. Previous national studies have generally reported positive attitudes toward AI among nurses [14, 69, 70], while an international meta‐analysis found that approximately 63% of nurses had positive attitudes [58]. AI attitudes have also been associated with CSE and innovation‐related outcomes [1, 71, 72]. In this study, AIASN was strongly correlated with CSES (*r* = 0.61) and perceived clinical competence (*r* = 0.65), while CSES was moderately correlated with perceived clinical competence (*r* = 0.47). The relationship reported by Şener, Güngör, and Taşdelen [69] between nurses’ individual innovativeness and attitudes toward AI, together with previous findings on AI and nurses’ innovative behavior [73], provides further support for the relationship between AI attitudes and innovation‐related individual characteristics. Overall, these findings suggest that more positive attitudes toward AI and higher CSE coexist with higher self‐reported clinical competence. However, the study did not assess actual clinical performance, clinical decision‐making, quality of care, or patient outcomes. Therefore, these findings refer to perceived clinical competence rather than objectively measured performance.

SCI was positively associated with AIASN (*r* = 0.42), CSES (*r* = 0.34), and perceived clinical competence (*r* = 0.42). These findings suggest that nurses’ perceptions of their clinical competence are associated with both individual characteristics and their perceived organizational environment. Previous nursing research has similarly reported positive relationships among organizational climate for innovation, CSE, and nurses’ innovative behavior [24, 71]. Although these studies examined innovative behavior rather than perceived clinical competence, they support considering individual and organizational factors concurrently when interpreting innovation‐related outcomes. Previous research also indicates that managerial support is associated with innovative behavior and other innovation‐related outcomes among nurses [74]. Accordingly, organizational conditions may be relevant to how individual attitudes and psychological resources are associated with nurses’ perceptions of their clinical competence.

The mediation analysis showed that AIASN was directly and indirectly associated with perceived clinical competence through SCI. Positive attitudes toward AI were associated with higher SCI scores, which were in turn associated with higher perceived clinical competence. The significant direct and indirect associations were consistent with partial statistical mediation. The indirect association became smaller after adjustment for age, gender, previous AI‐related training, previous AI use, educational level, professional experience, and institution type but remained statistically significant. This indicates that the indirect association was not fully explained by the selected covariates. SCI may therefore partly explain the relationship between AI attitudes and perceived clinical competence; however, this statistical mediation should not be interpreted as evidence of a causal process. Studies examining the same combination of variables remain limited [1]. Liu et al. [71] showed that an individual competence could alter the relationship between CSE and innovative behavior, although the moderation reported in that study was positive, whereas the interaction observed in this study was negative. AI self‐efficacy has also been reported to moderate the relationship between an evidence‐based practice climate and creativity‐supporting behaviors among nurse educators [75]. Although these studies examined different populations, variables, and outcomes, they suggest that individual competencies and organizational conditions may operate jointly.

The moderation analysis showed that the positive relationship between CSES and perceived clinical competence was stronger at lower levels of SCI and weaker at higher levels of SCI. When SCI is low, nurses may receive less managerial guidance, fewer organizational resources, and limited support for trying new approaches. Under these conditions, nurses with higher CSES may be more likely to take initiative, persist when problems arise, and develop alternative solutions. Nurses with lower CSES may have fewer personal and organizational resources on which to rely, potentially increasing the difference in perceived clinical competence between nurses with higher and lower CSES. In contrast, when SCI is high, shared resources, managerial support, and team‐based problem‐solving may support nurses across different levels of CSES. These organizational resources may reduce the extent to which perceived clinical competence depends on individual creative confidence. The interaction remained statistically significant after adjustment for demographic, professional, and AI‐related variables, indicating that the same pattern was observed in the adjusted model. A similar person–context interaction has been reported in research examining AI literacy and self‐efficacy among nursing students, although the population, variables, and outcomes differed from those examined in this study [76].

In the unadjusted simple slopes analysis, the relationship between CSES and perceived clinical competence was not statistically significant at an SCI score of 54.00 (Table 5), consistent with the gradual weakening of this relationship at higher SCI levels. This pattern can be interpreted from several complementary theoretical perspectives. Social Cognitive Theory proposes that individual beliefs and environmental conditions jointly shape behavior [77], while the Componential Theory of Creativity emphasizes the joint contribution of individual components and the social environment to creativity‐related outcomes [78]. Reviews of workplace creativity similarly highlight the combined role of personal and contextual characteristics [79, 80]. More specifically, situational strength theory proposes that clearer and more consistent expectations, support, and behavioral guidance may reduce the extent to which individual differences are expressed in outcomes [81]. Applied to these findings, a strong innovation climate may provide a more consistent organizational context in which nurses across different levels of CSES receive support and guidance. In contrast, when SCI is low, fewer shared resources and less consistent guidance may make CSE more relevant to perceived clinical competence. Because situational strength was not directly measured in this study, this explanation remains theoretical and requires further investigation. Importantly, the negative interaction does not indicate that a strong innovation climate is unfavorable. SCI remained positively associated with perceived clinical competence in the adjusted model. Rather, the interaction indicates that the relationship between CSES and perceived clinical competence became weaker as SCI increased.

### 4.1. Limitations

This study has several limitations. First, its cross‐sectional design does not establish the temporal order of the variables or support causal conclusions. Positive attitudes toward AI and a supportive innovation climate may be associated with higher perceived clinical competence; however, nurses who perceive themselves as more competent may also report more positive attitudes toward AI and their organizations. Therefore, the mediation and moderation findings should be interpreted as statistical associations rather than causal mechanisms. Second, convenience sampling and online recruitment may have resulted in selection bias. Nurses who were more interested in AI, innovation, or digital technologies may have been more likely to participate. This may have contributed to higher reported AI use and more positive AI attitudes in the sample and limits the generalizability of the findings. Third, all main variables were measured using self‐report instruments in the same survey. This may have increased the observed associations because of social desirability, response consistency, or common method bias. Although Harman’s single‐factor test did not indicate a dominant single‐factor structure, it cannot rule out common method bias. The direction and magnitude of this potential bias could not be determined. In addition, CSFCN measured nurses’ perceptions of their clinical competence rather than actual clinical performance. Therefore, the findings cannot be generalized to objectively assessed clinical skills, quality of care, patient safety, or patient outcomes. Finally, the questionnaire did not distinguish whether generative AI was used for clinical, educational, or personal purposes. Residual confounding is also possible because factors, such as workload, staffing, leadership style, digital infrastructure, and institutional AI policies, were not assessed.

### 4.2. Recommendations for Further Research

Future studies should use longitudinal designs to examine the temporal relationships among AI attitudes, CSE, innovation climate, and perceived clinical competence. Intervention studies are also needed to evaluate whether AI education or organizational innovation programs improve AI literacy, CSE, perceived or objectively assessed clinical competence, and other relevant outcomes. Future research should include objective measures, such as observed clinical performance, clinical decision‐making, quality indicators, and patient outcomes. The context and purpose of AI use should be clearly defined by distinguishing clinical, educational, and personal use. Probability‐based sampling and comparative studies across different healthcare systems are recommended to improve generalizability. Future moderation analyses should also examine whether the observed interaction varies according to organizational resources, leadership, staffing, and digital maturity.

### 4.3. Implications for Policy and Practice

Nursing education programs may consider including AI literacy, ethical use, data privacy, algorithmic bias, and verification of AI‐generated information in undergraduate and postgraduate curricula. Healthcare organizations may also develop clear policies defining approved AI tools, appropriate uses, data‐protection requirements, and the need for professional review of AI‐generated outputs. Because this study did not evaluate an educational intervention, these strategies should not be interpreted as interventions proven to improve perceived or objectively measured clinical competence. Nurse managers may support innovation by providing access to appropriate resources, opportunities for team‐based learning, and guidance for the safe evaluation of AI tools. The moderation findings suggest that additional mentoring and structured opportunities for problem‐solving could be considered for nurses working in environments with limited organizational support. Such organizational initiatives could initially be implemented as monitored pilot programs and evaluated using objective outcomes, including clinical performance, patient safety, and quality of care.

The findings may also be relevant to nursing management in other countries. However, healthcare systems differ in their digital infrastructure, AI regulations, staffing, professional roles, and organizational support for innovation. Therefore, AI‐related policies and management strategies should be adapted to local needs and resources. In settings with limited digital resources, basic infrastructure, staff training, and managerial support may require greater attention. In more digitally developed settings, priorities may include AI governance, ethical oversight, and evaluation of clinical outcomes. Because the study was conducted in Türkiye using convenience sampling, the applicability of the findings to other healthcare systems should be tested in international studies.

## 5. Conclusion

This study found significant relationships among nurses’ attitudes toward AI, CSE, perceived organizational climate for innovation, and perceived clinical competence. SCI partially mediated the relationship between AIASN and perceived clinical competence and moderated the relationship between CSES and perceived clinical competence. These relationships remained significant after adjustment for selected demographic, professional, and AI‐related variables. The relationship between CSES and perceived clinical competence became weaker as SCI increased, suggesting that individual CSE may be more relevant when organizational support for innovation is limited. However, the cross‐sectional and self‐report design does not support causal conclusions or conclusions about actual clinical performance, quality of care, or patient outcomes. Longitudinal and intervention studies using objective outcome measures are needed to confirm these findings.

## Author Contributions

Ozge Karakaya Suzan: conceptualization, methodology, validation, investigation, data curation, formal analysis, writing–original draft preparation, writing–review and editing, visualization, supervision, and project administration. Uğur Gül: conceptualization, validation, investigation, data curation, formal analysis, writing–original draft preparation, writing–review and editing, supervision, and project administration. Oğuz Koyuncu: conceptualization, methodology, software, validation, investigation, data curation, writing–original draft preparation, writing–review and editing, visualization, supervision, and project administration. Canberk Akdeniz: conceptualization, methodology, software, validation, investigation, data curation, writing–original draft preparation, writing–review and editing, visualization, supervision, and project administration.

## Funding

The authors have nothing to report.

## Disclosure

All the authors read and approved the final version of the manuscript.

## Ethics Statement

This study was prepared with the approval of the Sakarya University Ethics Committee dated 29.12.2025 and numbered E‐61923333‐050.99‐546697. This study was conducted in accordance with the principles of the World Medical Association Declaration of Helsinki. Written informed consent was obtained from the nurses.

## Conflicts of Interest

The authors declare no conflicts of interest.

## Data Availability

The data that support the findings of this study are available upon request from the corresponding author. The data are not publicly available due to privacy or ethical restrictions.
